# The performance of prostate‐specific antigen density in long‐term prostate cancer‐mortality risk prediction

**DOI:** 10.1002/bco2.70250

**Published:** 2026-07-10

**Authors:** Sara Saliba, Anders Kjellman, Anna Thor, Ulf Norming, Anna Lantz, Ove Gustafsson, Per‐Olof Lundgren

**Affiliations:** ^1^ Department of Clinical Science, Technology and Intervention Karolinska Institute Stockholm Sweden; ^2^ Department of Urology Karolinska University Hospital Stockholm Sweden; ^3^ Karolinska Institute, Department of Clinical Science and Education Södersjukhuset Stockholm Sweden; ^4^ Department of Molecular Medicine and Surgery Karolinska Institute Stockholm Sweden

**Keywords:** cancer‐specific mortality, prostate cancer, PSA‐density, screening

## Abstract

**Objective:**

The objective of this study is to examine whether a baseline calculation of PSAD is associated with prostate cancer‐specific mortality after 30 years of follow‐up.

**Methods:**

One‐thousand seven‐hundred and seventy‐nine men were screened for prostate cancer with a PSA test, digital rectal exam (DRE) and ultrasound of the prostate (TRUS). Outcomes, that is, prostate cancer‐specific mortality, were extracted from the Swedish National Cause of Death Registry. Hazard ratios for cancer‐specific deaths were estimated using Cox proportional hazard regression model. The area under the receiver operating curve (AUC) was determined using logistic regression for men with a baseline PSA in the interval between 3.0 and 10.0 ng/ml.

**Results:**

PSAD was available for 1748 men. Median PSAD was 0.095 ng/ml/cm^3^ (IQR 0.073–0.14). The age‐adjusted hazard ratio for lethal prostate cancer in the cohort of men with PSA between 3.0 and 10.0 ng/ml was 10.1 (95% CI 2.4–42.2) if PSAD was ≥0.15 compared to <0.15.

A model including PSAD, palpation and ultrasound findings could distinguish lethal prostate cancer from non‐cases at 30 years of follow‐up with an AUC of 0.75.

**Conclusions:**

For men with PSA between 3 and 10 ng/ml, PSAD is a predictor of the long‐term risk for lethal prostate cancer. One limitation to this study is the lack of clinical data during follow‐up apart from cancer‐specific mortality.

## 1 INTRODUCTION

### 1.1 Screening for prostate cancer

Prostate cancer remains one of the most common causes of cancer‐related deaths worldwide.^1^ Efforts are made to develop programmes for early detection of prostate cancer—and subsequently reducing the cancer‐specific mortality.^2^, ^3^ The European randomized screening for prostate cancer trial (ERSPC) did report a cancer‐specific survival benefit in the intervention arm after 13 years, whereas the American PLCO did not. As follow up time for the ERSPC increases, the numbers needed to screen to prevent one case of prostate cancer‐specific mortality decreases. At 23 years of follow up, the numbers needed to screen was 456 as opposed to 628 after 16 years of follow up.^4^, ^5^ It is widely acknowledged that the PLCO trial was to a large extent thwarted by frequent opportunistic screening in the control group. This, of course, limits a trial's ability to detect a survival benefit.

More contemporary trials evaluating prostate cancer screening include PSA testing, MRI and biopsies according to preset algorithms.^6^ Given the improved but more extensive, and thereby resource consuming, screening algorithms, tools to eliminate a subcohort from further screening would be beneficial.

### 1.2 PSA as a biomarker

Over time, several biomarkers have been identified for prostate cancer, both to detect the disease in its early stages but also to monitor cancer progression.^7^, ^8^ For 35 years, prostate cancer‐specific antigen (PSA) has been the most widespread marker used worldwide to diagnose and monitor prostate cancer. A solid body of evidence indicates that a single PSA predicts the risk of lethal prostate cancer at any later stage in life.^9^, ^10^ Although PSA is an important diagnostic tool for prostate cancer, it is not specific for cancer, as elevated values of PSA can be seen in benign prostatic hyperplasia.^11^ In a recent nonsystematic review, Bratt et al.^12^ concluded that low mid‐life PSA values can be utilized in adjusting screening intervals.

PSA's ability to discriminate between BPH and prostate cancer is not optimal in the PSA grey zone, that is, at intermediate levels between 3 and 10 ng/ml.^13^ Data suggest that predicting the pathological stage and risk for biochemical recurrence (BR) after a radical prostatectomy is challenging if the total PSA was within the grey zone pre‐operatively.^14^

### 1.3 PSA density

PSA density (PSAD) is used to discriminate elevated serum levels of PSA due to benign prostatic hyperplasia from high serum levels caused by malignant disease.^6^ Several studies have demonstrated an association between PSAD and risk for both biochemical recurrences after treatment with curative intent and the risk for upstaging after prostatectomy after an initial strategy of active monitoring.^15^, ^16^ In a prospective multicenter clinical trial, Magheli et al.^16^ found that PSAD was highly associated with pathological stage and biochemical recurrences post‐radical prostatectomy. PSAD was superior to total PSA in predicting biochemical recurrences (BR) in low‐grade prostate cancer.

PSAD is frequently used in clinical decision‐making where an elevated PSAD can lead to further diagnostic work up with MRI or biopsies, whereas a low PSAD in a man with relatively high PSA prompts a more conservative strategy.

Data regarding the ability of PSAD to discriminate between cases of lethal prostate cancer and non‐lethal cases are scarcer.

Pylväläinen et al.^17^ analysed a cohort of men with benign biopsies and could conclude that men with PSAD below 0.015 ng/ml/cm^3^ had a significantly lower cancer‐specific mortality than men with PSAD ≥ 0.015 ng/ml/cm^3^ after a median follow up of 17.6 years.

We aimed to assess the association between baseline PSAD and prostate cancer‐specific mortality after 30 years of follow up in a population‐based cohort of men aged between 55 and 70 years.

## 2 METHODS

This is a secondary analysis of the Stockholm screening for prostate cancer trial.^18^, ^19^ During the years 1988–1989, 2400 men between the ages of 55 and 70 were randomly sampled from a background population of about 27 000 men in the same age group living in a defined area of Stockholm, Sweden. The sampled group was invited to a screening study for prostate cancer including PSA, digital rectal examination (DRE) and TRUS.^14^ The TRUS included measurement of prostate size using the formula for the volume of an ellipse (H × W × L × 0.55).^20^ Of 2400 invited men, 1779 (74%) accepted the invitation and were thus screened for prostate cancer according to the algorithm. A subset of patients with baseline PSA between 3.0 and 10.0 ng/ml was further evaluated.

Prostate size was estimated using ultrasound by three experienced urologists.^18^ Concentration of PSA was estimated using the Hybritech tandem method.^21^ PSAD was then determined using the formula total PSA/prostate size = (ng/ml)/cm^3^.

All men regardless of PSA were examined with ultrasound and TRUS regardless of baseline PSA. The investigators were blind to the PSA value. Hence, in this analysis, the control group is the men with PSA between 3 and 10 ng/ml and PSAD below cut‐off. The screening effort itself yielded 65 cases of prostate cancer of which approximately one third was confined to the peripheral zone, 60% were grossly present but limited to the prostate gland and 9% exhibited extra prostatic growth corresponding to stage one, two and three of the, at the time, utilized staging algorithm.^22^ As for the interval cases, we lack clinical details beyond diagnosis and cause of death.

It is mandatory by law that the responsible physician must report a Swedish citizens death to the tax authority within 24 h. Furthermore, the cause of death, determined either by physical examination or by autopsy, must subsequently be reported within 3 weeks to the National Board of Health and Welfare and thereby be included in the National Cause of Death Registry. Outcomes for this study, that is, prostate cancer specific mortality, were extracted from the Swedish National Cause of Death Registry. The Swedish Cause of Death Registry is validated and assessed to be accurate and with a good coverage. However, it does to some extent, exaggerate malignant diseases impact on mortality.^23^

### 2.1 Statistical analysis

**FIGURE 1:**
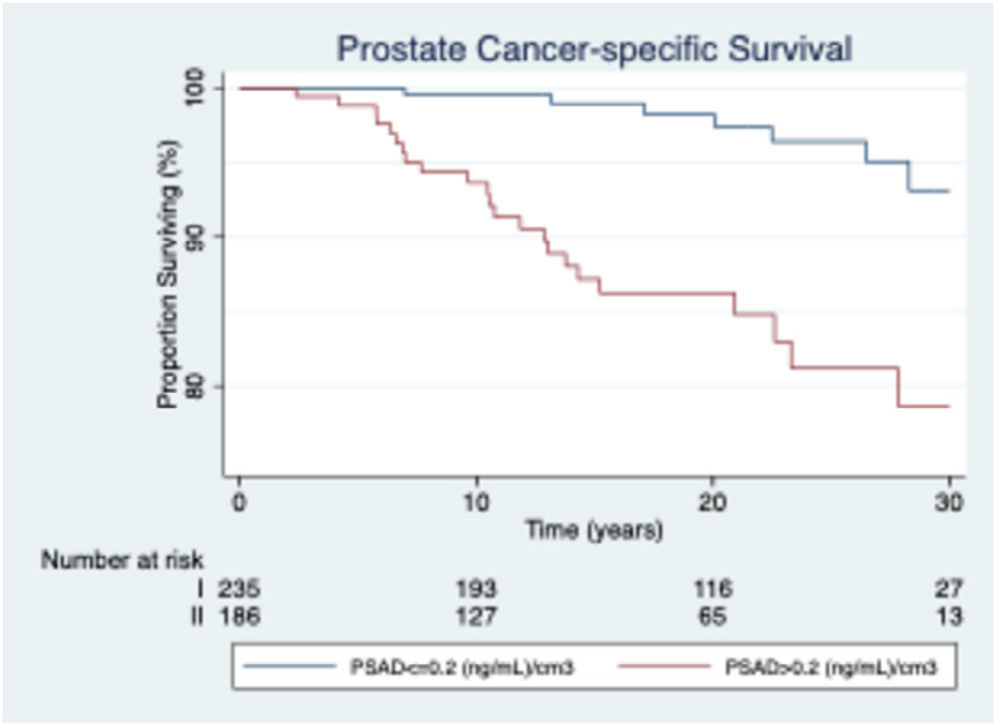
Kaplan Meier estimates of cancer‐specific survival stratified by PSAD above or below 0.2 (ng/ml)/cm^3^.

**FIGURE 2:**
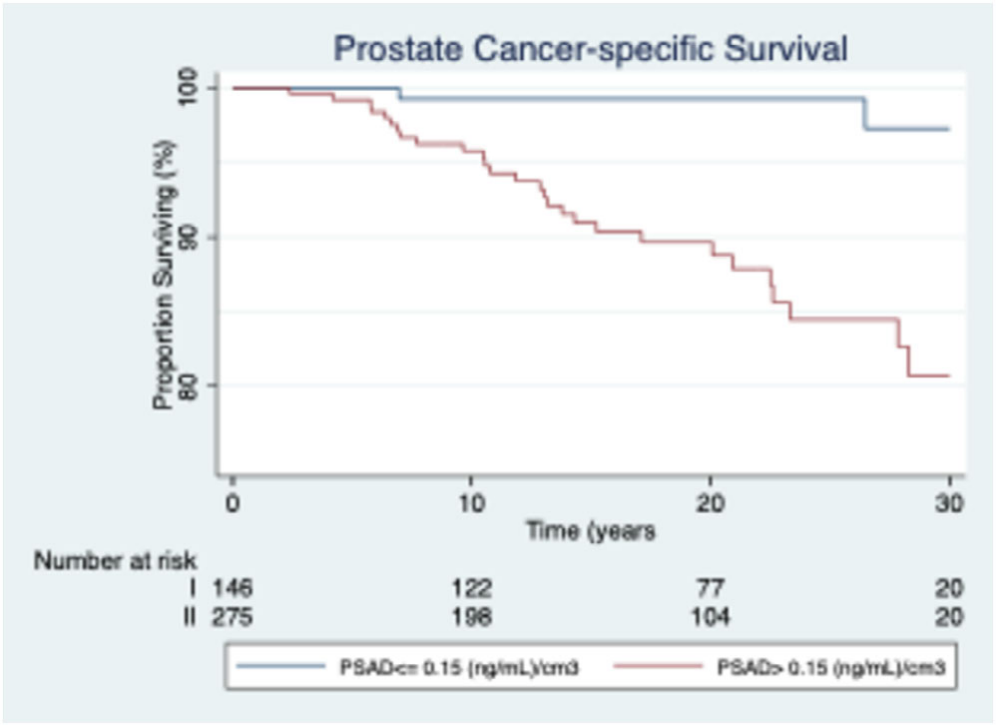
Kaplan Meier estimates of cancer‐specific survival stratified by PSAD above or below 0.15 (ng/ml)/cm^3^.

Follow‐up began on the day of screening and ended on the date of death or 31 December 2018. Estimations of association were modelled for the subcohort with PSA between 3.0 and 10 ng/ml. Two established cut‐off levels for PSAD were evaluated, that of current recommendation for MRI^24^ of 0.15 ng/ml/cm^3^ and the cut‐off for suspecting a significant cancer at 0.2 (ng/ml)/cm^3^.^25^ Also, the exploratory cut‐off levels of 0.1 ng/ml/cm^3^ and the median PSAD level for the entire cohort 0.095 (ng/ml)/cm^3^ was tested. Association between PSAD and cancer‐specific death was estimated using the Cox Proportional Hazard regression model. Assumption of proportionality tested with Schoenfeld residuals (*p* = 0.98 for the age adjusted model). A model including PSAD, DRE and ultrasound findings was developed using logistic regression. Its ability to distinguish cases of lethal prostate cancer from non‐cases was assessed using the area under ROC curve (AUC). For descriptive purposes, Kaplan–Meier estimates were calculated and graphed for survival probability (Figures 1 and 2). Differences were considered statistically significant if *p* < 0.05. All statistical analyses were made using STATA 16 software, College Station, Texas, United States.

The manuscript was drafted using the STROBE guidelines (strengthening the reporting of observational data).^26^ Ethical approval was obtained from the Swedish Regional Ethical Review Board (2017/1976‐32).

## 3 RESULTS

The entire cohort was evaluated after 30 years. After censoring for death from other causes and death from prostate cancer (that is failure in our survival analysis), median follow‐up was 18.5 years (IQR 11–27 years). In the entire cohort, 1294 died from other causes, and 88 died from lethal prostate cancer. Baseline characteristics of all men participating in the screening trial and for the subset of patients with PSA in the interval 3–10 ng/ml are provided in Table 1. In total, 421 men (24%) had a baseline PSA between 3.0 and 10 ng/ml with a median PSAD of 0.16 (IQR 0.12 to 0.22). Hazard ratio for prostate‐cancer specific mortality if PSAD was above 0.15 or 0.2 was 10.1 (2.4–42.2) and 5.5 (2.4–12.8), respectively.

**TABLE 1:** Baseline characteristics of the study cohort.

	*n*	PSA ng/mL, mean (range)	Prostate size cm^3^, mean (range)	Age, years, median (IQR)	Diagnosed with prostate cancer at end of follow up, *n* (%)	Alive at end of follow up, *n* (%)
Entire population	1779	3.3 (0.1–100)	24 (18–132)	64 (60–67)	262 (15)	396 (22)
Men with PSA 3.0–10.0 ng/ml	421	4.9 (3.0–9.9)	32 (19–108)	65(61–68)	99 (26)	69 (18)

Absolute risk for lethal prostate cancer was 5.5% if PSAD was less than 0.15 and 6.9% if PSAD was below 0.2 ng/ml/cm^3^. If PSAD was dichotomized by its median value for the entire cohort (0.095 ng/ml/cm^3^), there was no case of lethal prostate cancer in the group with PSAD below median.

All age adjusted and crude hazard ratios for lethal prostate cancer using different cut‐off levels of PSAD are presented in Table 2. Additionally, AUC for the three‐feature model (ultrasound, DRE and PSAD) for discriminating between cases and non‐cases is presented in the same table.

**TABLE 2:** Crude and age‐adjusted hazard ratios for prostate cancer‐specific death.

PSA‐density cut‐off (number of men above cut‐off)	Crude HR (95% CI)	*p*	Age adjusted HR (95% CI)	*p*	AUC^a^	Absolute risk for lethal prostate cancer *under* PSAD cut off (%)	Absolute risk for lethal prostate cancer *over* PSAD cut off (%)
0.2 (ng/ml)/cm^3^ (124)	5.6 (95% CI 2.4–13.1)	<0.0001	5.5 (2.4–12.8)	<0.0001	0.74	7.2	14.5
0.15 (ng/ml)/cm^3^ (161)	9.9 (2.4–41.9)	0.002	10.1 (2.4–42.2)	0.002	0.75	5.5	11.8
0.1 (ng/ml)/cm^3^ (326)	4.1 (2.1–8.1)	<0.0001	4.0 (2.0–7.9)	<0.0001	0.69	2.0	7.2
0.095 (ng/ml)/cm^3^ (334)	4.3 (2.1–9.0)	<0.0001	4.0 (2.0–7.9)	<0.0001	0.71	No events	7.2

^a^ Area under the curve (AUC) for a model including the PSAD‐levels, DRE and ultrasound and absolute risks for lethal prostate cancer for men with baseline PSA between 3.0 and 10.0 ng/ml.

The screening effort itself yielded 65 cases of prostate cancer. They were treated according to stage and contemporary practices; 11 were treated with radical prostatectomy, 26 with radiotherapy, 4 were included in a therapeutic study with YAG laser ablation, 18 were assigned to active surveillance, 5 were surgically castrated and 1 received androgen deprivation therapy.

## 4 DISCUSSION

### 4.1 Mortality rate estimation

Because prostate cancer is, in many cases, a slowly developing and progressing disease, assessment of biomarkers and predictors demands lengthy follow up. Management of long‐term data and risk assessment require statistical and methodological stringency. The more recent concepts of data mining and big data will likely prompt data driven decision making in time.^27^ Krasujalis et al.^28^ estimated mortality rates in a cohort of 2400 men treated with radical prostatectomy. After a median follow up of 7 years, the authors could conclude not only that lymph node status and pathological stage predict disease‐specific mortality but also that a Cox proportional hazard model was more accurate than a Fine‐Grey model in predicting risk. Another concern, and a possible limitation of this study, is the measurement of prostate volume—in this cohort, size estimation solely relies on TRUS. With contemporary standards, this is likely somewhat outdated as MRI estimations are increasingly considered gold standard for prostate volume measurement. Choe et al.^29^ compared results from volume measurements in 640 men and concluded that TRUS underestimates prostate size by about 8%. In a clinical setting, these differences will likely be of lesser concern, especially in a likely future with more data driven decision‐making aids. Also, all ultrasound size estimations were performed by two experienced urologists, likely improving reproducibility of the volume calculations.^18^

Attempts to predict negative events from prostate cancer typically focus on biochemical recurrences, presence of high‐grade cancer in biopsies or surgical specimens, or skeletal‐related events. A recent review of MRI‐based radiomics combined with clinical data could predict BR with an AUC of 0.88. Similarly, the use of the STHLM 3 panel in the re‐screening setting performed with an AUC of 0.765 for detecting significant cancer (Gleason ≥ 7),^30^ whereas MRI combined with prostate health index, fraction free PSA and PSAD reached an AUC of 0.885 for discriminating clinically significant cancer from seemingly indolent cases.^31^ Considering the increased use of MRI in prostate cancer work‐up, it is also reasonable to assume that volume estimations and hence PSAD estimations have improved.^32^

In the European guidelines, PSA is used for risk stratification regarding prevalence of clinically significant cancer and as a decision‐making tool whether to proceed with biopsies or not.^33^ Evidence regarding PSAD and long‐term risk assessment is scarcer. Tosoian at al.^34^ could conclude a higher risk for disease progression in men with high PSAD already designated to active surveillance after 5 years of follow up. Given PSA's ability to predict lifetime prostate cancer mortality risk, it is reasonable to evaluate PSAD for the same outcome. This study confirms that PSAD is a strong predictor of lifetime risk for non‐indolent prostate cancer. In addition, PSAD offers a non‐invasive and relatively cheap test compared to, for instance, MRI or the Stockholm 3 test.

Because all modern screening efforts aim to mitigate the risk of harm from overdiagnosis and overtreatment of prostate cancer, further methods to distinguish men at risk from those who are not are essential. The main benefit from this type of long‐term evaluation reasonably lies in its ability to aid in developing screening algorithms and long‐term risk assessment tools rather than aiding in the everyday clinical decision making.

One of the limitations of this study is the lack of clinical data other than cause of death. This may lead to an underestimation of clinically significant cancers. We do know, however, that metastasis is usually associated with cancer‐specific death, which is why this effect should be limited, that is, men with metastasized prostate cancer who dies is very likely to be assessed as prostate cancer‐specific mortality regardless of actual cause of death. In addition, we lack information about treatment of the interval cases—that is, the cases diagnosed after the initial screening. Strengths of this study include the mature nature of the cohort. With 30 years of follow‐up, few additional cases are expected.

## 5 CONCLUSIONS

PSAD offers a readily available, cheap and high‐performing alternative for long‐term prostate cancer‐specific survival prediction. As all screening for disease and prediction models for disease strive to detect significant disease while keeping over‐diagnosing and subsequent over‐treatment to a minimum, all practical tools to discriminate men at very low risk from those possibly benefiting from continuous monitoring are needed. PSA density proves to be a robust long‐term predictor for lethal prostate cancer. In this material, there were no cases of prostate‐cancer death in the group of men with PSA density below 0.095 ng/ml/cm^3^, indicating that these men can be excluded from further testing.

## AUTHOR CONTRIBUTIONS

*Study concept and design*: Ulf Norming, Ove Gustafsson, and Per‐Olof Lundgren. *Data Acquisition*: Ove Gustafsson, Ulf norming, and Per‐Olof Lundgren. *Data analysis and interpretation*: Sara Saliba, Anna Lantz, Anna Thor, Anders Kjellman, and Per‐Olof Lundgren. *Statistical analysis*: Anna Thor, Anna Lantz, Anders Kjellman, and Per‐Olof Lundgren. *Drafting of manuscript*: Sara Saliba and Per‐Olof Lundgren. Critical revision of manuscript: All authors.

## CONFLICT OF INTEREST STATEMENT

The authors declare that they have no competing financial interests or personal relationships that could have appeared to influence the work reported in this paper.

## Data Availability

The data that support the findings of this study are available on request from the corresponding author. The data are not publicly available due to privacy or ethical restrictions.

## References

[1] Siegel RL , Miller KD , Fuchs HE , Jemal A . Cancer statistics, 2021. CA Cancer J Clin. 2021;71(1):7–33. 10.3322/caac.21654 33433946

[2] Schroder FH , Hugosson J , Roobol MJ , Tammela TL , Zappa M , Nelen V , et al. Screening and prostate cancer mortality: results of the European Randomised Study of Screening for Prostate Cancer (ERSPC) at 13 years of follow‐up. Lancet. 2014;384(9959):2027–2035. 10.1016/S0140-6736(14)60525-0 25108889 PMC4427906

[3] Andriole GL , Levin DL , Crawford ED , Gelmann EP , Pinsky PF , Chia D , et al. Prostate cancer screening in the prostate, lung, colorectal and ovarian (PLCO) cancer screening trial: findings from the initial screening round of a randomized trial. J Natl Cancer Inst. 2005;97(6):433–438. 10.1093/jnci/dji065 15770007

[4] Hugosson J , Roobol MJ , Mansson M , Tammela TL , Zappa M , Nelen V , et al. A 16‐yr follow‐up of the European randomized study of screening for prostate cancer. Eur Urol. 2019;76(1):43–51. 10.1016/j.eururo.2019.02.009 30824296 PMC7513694

[5] Roobol MJ , de Vos II , Månsson M , Godtman RA , Talala KM , den Hond E , et al. European study of prostate cancer screening—23‐year follow‐up. N Engl J Med. 2025;393(17):1669–1680. 41160819 10.1056/NEJMoa2503223

[6] Arvendell M , Phillips L , Delilovic S , Enelius MB , Olsson K , Bolejko A , et al. Men's attitudes towards participation in organised prostate cancer testing: an abductive thematic analysis. Eur Urol Open Sci. 2025;71:156–164. 10.1016/j.euros.2024.12.007 39834916 PMC11743549

[7] Vickers AJ , Gupta A , Savage CJ , Pettersson K , Dahlin A , Bjartell A , et al. A panel of kallikrein marker predicts prostate cancer in a large, population‐based cohort followed for 15 years without screening. Cancer Epidemiol Biomarkers Prev. 2011;20(2):255–261. 10.1158/1055-9965.EPI-10-1003 21148123 PMC3035761

[8] Sartori DA , Chan DW . Biomarkers in prostate cancer: what's new? Curr Opin Oncol. 2014;26(3):259–264. 10.1097/CCO.0000000000000065 24626128 PMC4110681

[9] Mediu R , Rama A , Mediu N . Screening for prostate cancer: a study on the free and total prostate specific antigen. Discoveries (Sofia). 2021;9(4):e139. 10.15190/d.2021.18 PMC8960002 35359347

[10] Lundgren PO , Kjellman A , Norming U , Gustafsson O . Association between one‐time prostate‐specific antigen (PSA) test with free/total PSA ratio and prostate cancer mortality: a 30‐year prospective cohort study. BJU Int. 2021;128(4):490–496. 10.1111/bju.15417 33811738

[11] Collins GN , Lee RJ , McKelvie GB , Rogers AC , Hehir M . Relationship between prostate specific antigen, prostate volume and age in the benign prostate. Br J Urol. 1993;71(4):445–450. 10.1111/j.1464-410x.1993.tb15990.x 7684650

[12] Bratt O , Auvinen A , Arnsrud Godtman R , Hellström M , Hugosson J , Lilja H , et al. Screening for prostate cancer: evidence, ongoing trials, policies and knowledge gaps. BMJ Oncol. 2023;2(1):e000039. 10.1136/bmjonc-2023-000039 PMC11203092 39886507

[13] Benson MC , Whang IS , Pantuck A , Ring K , Kaplan SA , Olsson CA , et al. Prostate specific antigen density: a means of distinguishing benign prostatic hypertrophy and prostate cancer. J Urol. 1992;147(3 Pt 2):815–816. 10.1016/s0022-5347(17)37393-7 1371554

[14] Stamey TA , Johnstone IM , McNeal JE , Lu AY , Yemoto CM . Preoperative serum prostate specific antigen levels between 2 and 22 ng./ml. correlate poorly with post‐radical prostatectomy cancer morphology: Prostate specific antigen cure rates appear constant between 2 and 9 ng./ml. J Urol. 2002;167(1):103–111. 10.1016/s0022-5347(05)65392-x 11743285

[15] Freedland SJ , Wieder JA , Jack GS , Dorey F , deKernion JB , Aronson WJ . Improved risk stratification for biochemical recurrence after radical prostatectomy using a novel risk group system based on prostate specific antigen density and biopsy Gleason score. J Urol. 2002;168(1):110–115. 10.1016/s0022-5347(05)64841-0 12050502

[16] Magheli A , Rais‐Bahrami S , Trock BJ , Humphreys EB , Partin AW , Han M , et al. Prostate specific antigen versus prostate specific antigen density as a prognosticator of pathological characteristics and biochemical recurrence following radical prostatectomy. J Urol. 2008;179(5):1780–1784. 18343439 10.1016/j.juro.2008.01.032 PMC2675005

[17] Pylvalainen J , Talala K , Raitanen J , Rannikko A , Auvinen A . Association of prostate‐specific antigen density with prostate cancer mortality after a benign systematic prostate biopsy result. BJU Int. 2025;135(5):841–850. 10.1111/bju.16641 39840544 PMC11975165

[18] Gustafsson O , Norming U , Almgard LE , Fredriksson Å , Gustavsson G , Harvig B , et al. Diagnostic methods in the detection of prostate cancer: a study of a randomly selected population of 2,400 men. J Urol. 1992;148(6):1827–1831. 10.1016/S0022-5347(17)37041-6 1279225

[19] Tornblom M , Norming U , Adolfsson J , Becker C , Abrahamsson PA , Lilja H , et al. Diagnostic value of percent free prostate‐specific antigen: retrospective analysis of a population‐based screening study with emphasis on men with PSA levels less than 3.0 ng/mL. Urology. 1999;53(5):945–950. 10.1016/S0090-4295(98)00640-2 10223488

[20] Aprikian S , Luz M , Brimo F , Scarlata E , Hamel L , Cury FL , et al. Improving ultrasound‐based prostate volume estimation. BMC Urol. 2019;19(1):68. 10.1186/s12894-019-0492-2 31340802 PMC6657110

[21] Woodrum DL , French CM , Hill TM , Roman SJ , Slatore HL , Shaffer JL , et al. Analytical performance of the Tandem‐R free PSA immunoassay measuring free prostate‐specific antigen. Clin Chem. 1997;43(7):1203–1208. 10.1093/clinchem/43.7.1203 9216457

[22] Hermanek P , Scheibe O , Spiessl B , Wagner G . TNM classification of malignant tumors: the new 1987 edition. Chirurg. 1987;58(3):182. 10.1055/s-2007-1020216 3581988

[23] Brooke HL , Talback M , Hornblad J , Johansson LA , Ludvigsson JF , Druid H , et al. The Swedish cause of death register. Eur J Epidemiol. 2017;32(9):765–773. 10.1007/s10654-017-0316-1 28983736 PMC5662659

[24] Cornford P , van den Bergh RCN , Briers E , van den Broeck T , Brunckhorst O , Darraugh J , et al. EAU‐EANM‐ESTRO‐ESUR‐ISUP‐SIOG guidelines on prostate cancer‐2024 update. Part I: screening, diagnosis, and local treatment with curative intent. Eur Urol. 2024;86(2):148–163. 10.1016/j.eururo.2024.03.027 38614820

[25] Busch J , Hamborg K , Meyer HA , Buckendahl J , Magheli A , Lein M , et al. Value of prostate specific antigen density and percent free prostate specific antigen for prostate cancer prognosis. J Urol. 2012;188(6):2165–2170. 10.1016/j.juro.2012.07.106 23083861

[26] von Elm E , Altman DG , Egger M , Pocock SJ , Gøtzsche PC , Vandenbroucke JP . The Strengthening the Reporting of Observational Studies in Epidemiology (STROBE) statement: guidelines for reporting observational studies. Lancet. 2007;370(9596):1453–1457. 10.1016/S0140-6736(07)61602-X 18064739

[27] Subrahmanya SVG , Shetty DK , Patil V , Hameed BMZ , Paul R , Smriti K , et al. The role of data science in healthcare advancements: applications, benefits, and future prospects. Irish J Med Sci. 2022;191(4):1473–1483. 10.1007/s11845-021-02730-z 34398394 PMC9308575

[28] Kraujalis V , Ruzgas T , Milonas D . Mortality rate estimation models for patients with prostate cancer diagnosis. Balt J Mod Comput. 2022;10(2):170–184.

[29] Choe S , Patel HD , Lanzotti N , Okabe Y , Rac G , Shea SM , et al. MRI vs transrectal ultrasound to estimate prostate volume and PSAD: impact on prostate cancer detection. Urology. 2023;171:172–178. 10.1016/j.urology.2022.09.007 36152871

[30] Discacciati A , Abbadi A , al Clements MS e , Annerstedt M , Carlsson S , Grönberg H , et al. Repeat prostate cancer screening using blood‐based risk prediction or prostate‐specific antigen in the era of magnetic resonance imaging‐guided biopsies: a secondary analysis of the STHLM3‐MRI randomized clinical trial. Eur Urol Oncol. 2024;8(6):1466–1473. 10.1016/j.euo.2024.10.016 39562218

[31] Siddiqui MR , Li EV , Kumar S , al Busza A e , Kumar SKSR , Lin JS , et al. Optimizing detection of clinically significant prostate cancer through nomograms incorporating MRI, clinical features, and advanced serum biomarkers in biopsy naive men. Prostate Cancer Prostatic Dis. 2023;26(3):588–595. 10.1038/s41391-023-00660-8 36973367

[32] Patel HD , Koehne EL , Shea SM , Fang AM , Gerena M , Gorbonos A , et al. A prostate biopsy risk calculator based on MRI: development and comparison of the Prospective Loyola University multiparametric MRI (PLUM) and Prostate Biopsy Collaborative Group (PBCG) risk calculators. BJU Int. 2023;131(2):227–235. 10.1111/bju.15835 35733400 PMC9772358

[33] Schoots IG , Padhani AR . Risk‐adapted biopsy decision based on prostate magnetic resonance imaging and prostate‐specific antigen density for enhanced biopsy avoidance in first prostate cancer diagnostic evaluation. BJU Int. 2021;127(2):175–178. 10.1111/bju.15277 33089586 PMC7894174

[34] Tosoian JJ , Mamawala M , Epstein JI , Landis P , Macura KJ , Simopoulos DN , et al. Active surveillance of grade Group 1 prostate cancer: long‐term outcomes from a large prospective cohort. Eur Urol. 2020;77(6):675–682. 10.1016/j.eururo.2019.12.017 31918957

